# No Major Effect of Innate Immune Genetics on Acute Kidney Rejection in the First 2 Weeks Post-Transplantation

**DOI:** 10.3389/fphar.2019.01686

**Published:** 2020-02-20

**Authors:** Rong Hu, Daniel T. Barratt, Janet K. Coller, Benedetta C. Sallustio, Andrew A. Somogyi

**Affiliations:** ^1^ Discipline of Pharmacology, Adelaide Medical School, University of Adelaide, Adelaide, SA, Australia; ^2^ Department of Clinical Pharmacology, The Queen Elizabeth Hospital, Adelaide, SA, Australia; ^3^ Department of Clinical Pharmacology, Royal Adelaide Hospital, Adelaide, SA, Australia

**Keywords:** tacrolimus, immune genetics, kidney transplantation, acute rejection, *IL6* -6331

## Abstract

**Background:**

Innate immunity contributes to acute rejection after kidney transplantation. Genetic polymorphisms affecting innate immunity may therefore influence patients’ risk of rejection. *IL2* -330T > G, *IL10* -1082G > A, -819C > T, and -592C > A, and *TNF* -308G > A are not associated with acute rejection incidence in Caucasian kidney transplant recipients receiving a calcineurin inhibitor, ciclosporin or tacrolimus (TAC). However, other important innate immune genetic polymorphisms have not yet been extensively studied in recipients and donors. In addition, innate immunogenetics have not been investigated in kidney transplant cohorts receiving only TAC as the calcineurin inhibitor.

**Objective:**

To investigate the effect of recipient and donor *CASP1*, *CRP*, *IL1B*, *IL2*, *IL6*, *IL6R*, *IL10*, *MYD88*, *TGFB*, *TLR2*, *TLR4*, and *TNF* genetics on acute kidney rejection in the first 2 weeks post-transplant in TAC-treated kidney transplant recipients.

**Methods:**

This study included 154 kidney transplant recipients and 81 donors successfully genotyped for 17 polymorphisms in these genes. All recipients were under triple immunosuppressant therapy of TAC, mycophenolate mofetil, and prednisolone. Recipient and donor genotype differences in acute rejection incidence within the first 2 weeks post-transplantation were assessed by logistic regression, adjusting for induction therapy, human leukocyte antigen mismatches, kidney transplant number, living donor, and peak panel-reactive antibody scores.

**Results:**

A trend (Cochran-Armitage P = 0.031) of increasing acute rejection incidence was observed from recipient *IL6* -6331 T/T (18%) to T/C (25%) to C/C (46%) genotype [C/C versus T/T odds ratio (95% confidence interval) = 6.6 (1.7 to 25.8) (point-wise P = 0.017)]. However, no genotype differences were significant after Bonferroni correction for multiple comparisons.

**Conclusions:**

This study did not detect any statistically significant effects of recipient or donor innate immune genetics on acute rejection incidence in the first 2 weeks post-transplantation. However, the sample size was small, and future larger studies or meta-analyses are required to demonstrate conclusively if innate immune genetics such as *IL6* influence the risk of acute rejection after kidney transplantation.

## Introduction

Acute rejection is the major short-term challenge following kidney transplantation and it also increases long-term graft loss (McDonald et al., 2007). Although induction therapy, human leukocyte antigen (HLA) mismatches, number of kidney transplants, living donor, and peak panel-reactive antibodies (PRAs) have been studied as potential acute rejection predictors (Hammond et al., 2010; Lim et al., 2012; Lim et al., 2015; Zhu et al., 2016), these factors only contribute partially to acute rejection incidence.

While the T-cell driven adaptive immune system is essential to acute rejection, the innate immune system also plays a key role. Extracellular damage-associated molecular patterns from transplantation surgery and ischemia/reperfusion injury can induce the translocation of nuclear factor κ-light-chain-enhancer of activated B cells (NF-κB) into T-cell nuclei *via* activation of the myeloid differentiation primary response 88 (MyD88)-dependent Toll-like receptor (TLR) signaling pathway (Li and Verma, 2002; Liew et al., 2005). Translocated NF-κB activates pro-inflammatory cytokine secretion [e.g. pro-interleukin (IL)-1β, IL-2, and tumor necrosis factor-α (TNF-α)] (Li and Verma, 2002). Caspase 1 (encoded by *CASP1*) is an inflammatory response initiator and converts pro-IL-1β into mature IL-1β (Kostura et al., 1989; Thornberry et al., 1992). These pro-inflammatory mediators can assist T-cell activation, proliferation, and differentiation, and intensify kidney tissue damage (Watson et al., 1980; Nankivell and Alexander, 2010; Anders and Schaefer, 2014). In contrast, anti-inflammatory cytokines (e.g. IL-10) can decrease pro-inflammatory cytokine release (Walsh et al., 2004) and therefore have the potential to attenuate rejection risk, whereas transforming growth factor β (TGF-β) and IL-6 have both pro- and anti-inflammatory action (Saxena et al., 2008; Scheller et al., 2011). Notably, IL-6 trans-signaling *via* soluble IL-6 receptor (IL-6R) is pro-inflammatory as it can enhance the expansion and activation of T- and B-cells and induce several acute phase reactants such as C-reactive protein (CRP) (Wolf et al., 2014).

Single nucleotide polymorphisms (SNPs) in *CASP1*, *CRP*, *IL1B*, *IL2*, *IL6*, *IL6R*, *IL10*, *TGFB*, and *TNF* can increase or decrease the protein production and/or function of these pro- and anti-inflammatory mediators *in vitro* (Kroeger et al., 1997; Turner et al., 1997; Awad et al., 1998; Hoffmann et al., 2001; Dunning et al., 2003; Hall et al., 2004; Trompet et al., 2008; Wang et al., 2009) or serum/plasma concentrations *in vivo* (Fishman et al., 1998; Grainger et al., 1999; Galicia et al., 2004; Smith et al., 2008; Lacruz-Guzmán et al., 2013). In addition, SNPs in the MyD88-dependent TLR signaling pathway affect innate immune responses to vaccines (Ovsyannikova et al., 2011) and susceptibility to infection or disease *in vivo* (Taniguchi et al., 2013; Santos-Martins et al., 2014). Therefore, these innate immunogenetic markers may serve as predictors of acute rejection post-kidney transplantation.

Meta-analyses have shown that recipient and/or donor *IL2* -330T > G (rs2069762), *IL10* -1082G > A (rs1800896), -819C > T (rs1800871), and -592C > A (rs1800872), and *TNF* -308G > A (rs1800629) SNPs do not affect acute rejection incidence in Caucasian kidney transplant recipients receiving immunosuppressive therapy (Hu et al., 2011; Hu et al., 2015; Xiong et al., 2015; Hu et al., 2016). However, none of the cross-sectional studies included in these meta-analyses was carried out in recipients treated with tacrolimus (TAC) as the sole calcineurin inhibitor (CNI). Since TAC has potent immunosuppression 100 times greater than ciclosporin (Kino et al., 1987), with fewer rejection complications (U.S. Multicenter FK506 Liver Study Group, 1994), most kidney transplant recipients in Europe and Australia have been treated with TAC as the first-choice CNI for immunosuppression therapy since 2003 (Wadström et al., 2017) and 2009 (ANZDATARegistry, 2010), respectively. Therefore, it is worthwhile exploring the innate immunogenetic impact on kidney transplant recipients treated with only TAC as the CNI.

Only one study has investigated the impact of *IL1B* 3954C > T (rs1143634) on acute rejection incidence in kidney transplant recipients and found recipient 3954C/T genotype had higher rejection incidence than C/C genotype (point-wise P = 0.045) but without multiple comparison adjustment (Manchanda and Mittal, 2008). In terms of *TLR4* 896A > G (rs4986790) and 1196C > T (rs4986791), it is still controversial if these two SNPs affect acute rejection incidence in kidney transplant recipients (Ducloux et al., 2005; Palmer et al., 2006; Nogueira et al., 2007). Limited sample size, low minor allele frequency of the *TLR4* SNPs, different criteria for acute rejection [biopsy-proven acute rejection (BPAR) versus clinical evidence, e.g. serum creatinine change], varied recipient/donor ethnicities, and different time of rejection post-transplantation between cross-sectional studies may contribute together to the inconsistent findings of *TLR4* genetics on acute rejection incidence. In addition, adjustment for multiple statistical comparisons was not conducted. Notably, SNPs in *CASP1*, *CRP*, *IL6R*, *MYD88*, and *TLR2* have not been examined for their impact on acute rejection in kidney transplant recipients.

To bridge these research gaps, this study aimed to explore the impact of recipient and donor *CASP1*, *CRP*, *IL1B*, *IL2*, *IL6*, *IL6R*, *IL10*, *MYD88*, *TGFB*, *TLR2*, *TLR4*, and *TNF* genotypes on BPAR incidence in a cohort of predominantly Caucasian kidney transplant recipients treated with TAC as the only CNI (Hu et al., 2018; Hu et al., 2019a; Hu et al., 2019b). We hypothesized that these recipient and donor innate immunogenetics would affect BPAR incidence in kidney transplant recipients in the first 2 weeks post-transplantation.

## Materials and Methods

### Study Participants and Data Collection

This study was approved by the Central Adelaide Local Health Network Human Research Ethics Committee (Protocol number 2008178). All procedures complied with the Declaration of Helsinki and/or institutional research committee ethical requirements.

As described previously, 165 kidney transplant recipients and 129 donors were recruited (Hu et al., 2018; Hu et al., 2019a; Hu et al., 2019b). All recipients and living donors gave written informed consent before participation. For deceased donors, their respective recipients gave informed consent to use excess donor tissue blood vessels for genotyping. Recipient inclusion and exclusion criteria, demographics, anti-CD-25 induction therapy, immunosuppressant regimen (TAC, mycophenolate mofetil, and prednisolone), the number of HLA mismatches (HLA-A, -B, and -DR antigens) between recipients and donors, number of kidney transplants, donor type (living or deceased), PRA scores (%), and BPAR based on Banff classification of Solez et al., 2008 (as the transplants were performed between 2005 and 2011) have been described previously (Hu et al., 2018; Hu et al., 2019a; Hu et al., 2019b).

### Genotyping

Genomic DNA was extracted from blood, buccal swab, and kidney tissue (Hu et al., 2018; Hu et al., 2019a). A panel of 21 SNPs in 15 genes described previously (Mulholland et al., 2014; Barratt et al., 2015; Coller et al., 2015; Somogyi et al., 2016; Coller et al., 2019) were assayed using Agena Bioscience (formerly known as Sequenom) MassARRAY at the Australian Genome Research Facility (Brisbane, Australia). This panel included SNPs in the MyD88-dependent TLR signaling pathway—*TLR2* 1350T > C (rs3804100), *TLR4* 896A > G and 1196C > T, and *MYD88* 1593A > G (rs6853); pro- and anti-inflammatory mediators—*CASP1* 5352G > A (rs580253) and 10643G > C (rs554344), *CRP* -717T > C (rs2794521), *IL1B* -511C > T (rs16944), -31T > C (rs1143627), and -3954C > T, *IL2* -330T > G, *IL6* -6331T > C (rs10499563), *IL6R* -48892A > C (rs8192284), *IL10* -1082G > A and -819C > T, *TGFB* -509C > T (rs1800469), and *TNF* -308G > A. The panel also included *BDNF* 196G > A (rs6265) and *OPRM1* 118A > G (rs1799971) that were considered outside the scope of this study, and *TGFB* -1287G > A (rs11466314) and *LY96* 379C > T (rs11466004) that are known to be of very low frequency in Caucasians; these four SNPs were therefore not included in the analyses described below.

### Statistical Analyses

Hardy-Weinberg Equilibrium (HWE) tests for all genotypes, linkage disequilibrium (LD) between SNPs and haplotype inference within genes, and logistic regression analyses, were as described previously (Hu et al., 2018; Hu et al., 2019a). Due to the relatively limited sample size, only SNPs with minor allele frequencies >5% were included in logistic regression analyses. For SNPs in perfect or near-perfect (r^2^ > 0.9) LD, only 1 of the linked SNPs in that gene, instead of haplotypes/diplotypes, was analyzed in logistic regression analysis.

Genotype differences in BPAR incidence were analyzed for each SNP separately by logistic regression, adjusting for induction therapy [yes/no (Y/N)], living donor (Y/N), HLA mismatches (<3 or ≥3), kidney transplant number (1 or ≥ 2), and peak PRA scores (≤10% or >10%). Statistical significance was assessed by the likelihood-ratio test, and effects described by odds ratios (OR) with 95% confidence intervals (CI). Genotype differences in BPAR without adjusting for non-genetic variables were tested by Cochran-Armitage test for trend in GraphPad Prism v8 (GraphPad Software, San Diego, CA, USA), or Fisher’s exact test for SNPs with rare homozygous genotypes (n < 5) combined with heterozygotes, and OR with 95% CI.

P-value thresholds for significance were corrected for multiple testing by Bonferroni-adjustment (α = 0.05/N, where N is the number of SNPs analyzed in the recipient or donor cohort, respectively).

## Results

One hundred and fifty-four recipients and 81 (57 living, 24 deceased) donors had sufficient DNA for genotyping. In total, 23% (n = 35) of recipients with genotype data developed BPAR in the first 2 weeks post-transplantation. The impact of induction therapy, HLA mismatches, kidney transplant number, living donor, and peak PRA scores on BPAR incidence has been reported (Hu et al., 2019a); none were statistically significant (likelihood-ratio test P-value > 0.1).

### Genetic Variability in Kidney Transplant Recipients and Donors

All recipient and donor allele and genotype frequencies are summarized in **Table 1**. Six recipients each received a kidney from three deceased donors (two kidneys per donor), therefore, these three donors were counted only once for HWE tests but were treated independently for logistic regression analyses. For some SNPs, one to four recipients and/or donors had missing genotypes due to genotyping failure. All recipient and donor genotypes were in HWE (P ≥ 0.2). *CASP1*, *IL1B*, *IL10*, and *TLR4* haplotype and diplotype frequencies are summarized in **Supplementary Table 1**. Recipient and donor *CASP1* 10643G and 5352G, *IL1B* -511C and -31T, and *TLR4* 896A and 1196C were in perfect or near-perfect LD (D’ > 0.99; r^2^ ≥ 0.96) while *IL10* -1082G and -819C were in complete but not perfect LD [D’ = 1.0; r^2^ = 0.30; resulting in six observed diplotypes (**Supplementary Table 1**)]. Therefore, only 5352G > A in *CASP1*, -511C > T and 3954C > T in *IL1B*, and 896A > G in *TLR4*, along with all SNPs (including *IL10* -1082G > A and -819C > T separately) in other innate immune genes, were included in the subsequent analyses.

**Table 1:** Recipient and donor genotype and allele frequencies of SNPs in pro- and anti-inflammatory mediators and MyD88-dependent TLR signaling pathway genes.

Genes & SNPs		Recipients^#^ (n = 153–154)			Donors* (n = 77–81)		
		Genotypes (n, %)	Alleles (n, %)	HWE P	Genotypes (n, %)	Alleles (n, %)	HWE P
*CASP1*	5352G > A	G/G (107, 69)	G (258, 84)	0.8	G/G (58, 72)	G (137, 85)	1
		G/A (44, 29)	A (50, 16)		G/A (21, 26)	A (25, 15)	
		A/A (3, 2)			A/A (2, 2)		
	10643G > C	G/G (107, 69)	G (258, 84)	0.8	G/G (58, 72)	G (137, 85)	1
		G/C (44, 29)	C (50, 16)		G/C (21, 26)	C (25, 15)	
		C/C (3, 2)			C/C (2, 2)		
*CRP*	-717T > C	T/T (77, 50)	T (215, 70)	0.4	T/T (33, 41)	T (103, 64)	1
		T/C (61, 40)	C (93, 30)		T/C (37, 46)	C (57, 36)	
		C/C (16, 10)			C/C (10, 13)		
*IL1B*	-511C > T	C/C (76, 49)	C (215, 70)	0.7	C/C (41, 51)	C (114, 70)	0.8
		C/T (63, 41)	T (93, 30)		C/T (32, 40)	T (48, 30)	
		T/T (15, 10)			T/T (8, 10)		
	-31T > C	T/T (74, 48)	T (211, 69)	0.7	T/T (41, 51)	T (114, 70)	0.8
		T/C (63, 41)	C (95, 31)		T/C (32, 40)	C (48, 30)	
		C/C (16, 10)			C/C (8, 10)		
	3954C > T	C/C (84, 55)	C (229, 74)	0.5	C/C (52, 64)	C (128, 79)	0.5
		C/T (61, 40)	T (79, 26)		C/T (24, 30)	T (34, 21)	
		T/T (9, 6)			T/T (5, 6)		
*IL2*	-330T > G	T/T (70, 45)	T (203, 66)	0.3	T/T (39, 48)	T (114, 70)	0.6
		T/G (63, 41)	G (105, 34)		T/G (36, 44)	G (48, 30)	
		G/G (21, 14)			G/G (6, 7)		
*IL6*	-6331T > C	T/T (80, 52)	T (221, 72)	0.8	T/T (50, 62)	T (128, 79)	1
		T/C (61, 40)	C (87, 28)		T/C (28, 35)	C (34, 21)	
		C/C (13, 8)			C/C (3, 4)		
*IL6R*	48892 > C	A/A (50, 33)	A (178, 58)	0.6	A/A (27, 34)	A (93, 58)	1
		A/C (78, 51)	C (128, 42)		A/C (39, 49)	C (67, 42)	
		C/C (25, 16)			C/C (14, 18)		
*IL10*	-1082G > A	G/G (31, 20)	G (141, 46)	0.6	G/G (16, 20)	G (68, 42)	0.5
		G/A (79, 52)	A (165, 54)		G/A (36, 44)	A (94, 58)	
		A/A (43, 28)			A/A (29, 36)		
	-819C > T	C/C (88, 58)	C (230, 75)	0.5	C/C (42, 52)	C (119, 73)	0.4
		C/T (54, 35)	T (76, 25)		C/T (35, 43)	T (43, 27)	
		T/T (11, 7)			T/T (4, 5)		
*MYD88*	1593A > G	A/A (123, 80)	A (275, 89)	0.7	A/A (64, 79)	A (145, 90)	0.6
		A/G (29, 19)	G (33, 11)		A/G (17, 21)	G (17, 10)	
		G/G (2, 1)			G/G (0, 0)		
*TGFB*	-509C > T	C/C (81, 53)	C (222, 72)	0.8	C/C (45, 56)	C (119, 73)	0.6
		C/T (60, 39)	T (86, 28)		C/T (29, 36)	T (43, 27)	
		T/T (13, 8)			T/T (7, 9)		
*TLR2*	1350T > C	T/T (133, 86)	T (285, 93)	0.2	T/T (74, 91)	T (154, 95)	0.2
		T/C (19, 12)	C (23, 7)		T/C (6, 7)	C (8, 5)	
		C/C (2, 1)			C/C (1, 1)		
*TLR4*	896A > G	A/A (137, 89)	A (290, 94)	0.4	A/A (71, 88)	A (152, 94)	1
		A/G (16, 10)	G (18, 6)		A/G (10, 12)	G (10, 6)	
		G/G (1, 1)			G/G (0, 0)		
	1196C > T	C/C (136, 88)	C (289, 94)	0.4	C/C (70, 88)	C (150, 94)	1
		C/T (17, 11)	T (19, 6)		C/T (10, 13)	T (10, 6)	
		T/T (1, 1)			T/T (0, 0)		
*TNF*	-308G > A	G/G (113, 73)	G (261, 85)	0.2	G/G (50, 62)	G (130, 80)	0.2
		G/A (35, 23)	A (47, 15)		G/A (30, 37)	A (32, 20)	
		A/A (6, 4)			A/A (1, 1)		

HWE P, Hardy-Weinberg Equilibrium P-value; n, number; SNP, single nucleotide polymorphism.

Donors*: donor numbers may differ from those in **Table 2**, as 3 deceased donors each provided kidneys for 6 different recipients, these 3 donors were not counted twice in HWE; also, donor numbers may differ within **Table 1** due to genotyping failure.

Recipients ^#^: recipient numbers may differ within **Table 1** due to genotyping failure.

Rare homozygous genotypes (n < 5) were combined with heterozygous genotypes for logistic regression and Fisher’s exact test as follows: recipient *MYD88* rs6853 A/A genotype versus G allele carriers (A/G + G/G), *TLR4* 896A/A genotype versus G allele carriers (A/G + G/G); donor *IL6* -6331T/T genotype versus C allele carriers (T/C + C/C); recipient and donor *CASP1* 5352G/G genotype versus A allele carriers (G/A + A/A), *TLR2* 1350T/T genotype versus C allele carriers (T/C + C/C), *TNF* -308G/G genotype versus A allele carriers (G/A + A/A).

Consequently, a multiple testing-adjusted P-value threshold for significance was determined at 0.0036 (α = 0.05/14).

### Innate Immunogenetic Impact on BPAR Incidence

**Table 2** summarizes the associations between recipient and donor genotypes and BPAR incidence in the first 2 weeks post-transplantation, adjusting for induction therapy, HLA mismatches, kidney transplant number, living donor, and peak PRA scores. Although recipients with *IL6* -6331C/C genotype had a higher incidence of BPAR compared to T/T genotype recipients [OR (95% CI) = 6.6 (1.7–25.8), likelihood-ratio test P-value = 0.017], none of the genetic factors (including *IL6* -6331T > C) statistically significantly affected BPAR incidence after correction for multiple comparisons (P-value threshold = 0.0036).

**Table 2:** Recipient and Donor Innate Immune Genotype Differences in BPAR Incidence in the first 2 Weeks Post-Transplantation, Adjusting for HLA Mismatches, Induction Therapy, Kidney Transplant Number, Living Donor and Peak PRA Scores.

Genes & SNPs		Recipients^#^ (n = 153–154)				Donors* (n = 83–84)			
		Genotypes (n)	BPAR (n, %)	OR [95% CI]	P	Genotypes (n)	BPAR (n, %)	OR [95% CI]	P
*CASP1*	5352G > A	G/G (107)	19, 18	Ref	0.07	G/G (60)	16, 27	Ref	0.9
		G/A + A/A (47)	16, 34	2.2 [0.9–5.2]		G/A + A/A (24)	7, 29	1.0 [0.3–2.9]	
*CRP*	-717T > C	T/T (77)	12, 16	Ref	0.05	T/T (34)	6, 18	Ref	0.1
		T/C (61)	18, 30	3.0 [1.2–7.6]		T/C (39)	15, 38	3.1 [1.0–10.5]	
		C/C (16)	5, 31	2.1 [0.5–7.8]		C/C (10)	2, 20	1.3 [0.2–7.5]	
*IL1B*	-511C > T	C/C (76)	18, 24	Ref	0.9	C/C (41)	13, 32	Ref	0.5
		C/T (63)	13, 21	0.8 [0.3–1.9]		C/T (34)	9, 26	0.7 [0.2–2.2]	
		T/T (15)	4, 27	0.9 [0.2–3.6]		T/T (9)	1, 11	0.3 [0.01–1.9]	
	3954C > T	C/C (84)	16, 19	Ref	0.2	C/C (54)	13, 24	Ref	0.07
		C/T (61)	18, 30	2.0 [0.9–4.6]		C/T (25)	10, 40	2.3 [0.8–6.6]	
		T/T (9)	1, 11	0.6 [0.03–4.1]		T/T (5)	0, 0	NA	
*IL2*	-330T > G	T/T (70)	12, 17	Ref	0.3	T/T (41)	10, 24	Ref	0.09
		T/G (63)	16, 25	1.5 [0.6–3.6]		T/G (37)	9, 24	1.1 [0.4–3.2]	
		G/G (21)	7, 33	2.4 [0.7–7.2]		G/G (6)	4, 67	8.1 [1.2–78.5]	
*IL6*	-6331T > C	T/T (80)	14, 18	Ref	0.02	T/T (52)	11, 21	Ref	0.09
		T/C (61)	15, 25	1.6 [0.7–4.0]		T/C + C/C (32)	12, 38	2.4 [0.9–6.9]	
		C/C (13)	6, 46	6.6 [1.7–25.8]					
*IL6R*	48892A > C	A/A (50)	12, 24	Ref	0.9	A/A (29)	4, 14	Ref	0.09
		A/C (78)	16, 21	0.8 [0.3–2.1]		A/C (39)	11, 28	2.3 [0.6–10.1]	
		C/C (25)	6, 24	0.9 [0.3–3.2]		C/C (15)	7, 47	5.4 [1.2–27.5]	
*IL10*	-1082G > A	G/G (31)	8, 26	Ref	0.7	G/G (18)	3, 17	Ref	0.4
		G/A (79)	19, 24	1.0 [0.4–2.9]		G/A (37)	11, 30	2.3 [0.6–11.8]	
		A/A (43)	8, 19	0.7 [0.2–2.3]		A/A (29)	9, 31	2.5 [0.6–13.3]	
	-819C > T	C/C (88)	22, 25	Ref	0.4	C/C (44)	9, 20	Ref	0.05
		C/T (54)	10, 19	0.6 [0.2–1.4]		C/T (36)	14, 39	2.7 [1.0–7.9]	
		T/T (11)	3, 27	1.2 [0.2–4.6]		T/T (4)	0, 0	NA	
*MYD88*	1593A > G	A/A (123)	28, 23	Ref	0.6	A/A (66)	17, 26	Ref	0.5
		A/G + G/G (31)	7, 23	0.7 [0.2–2.0]		A/G (18)	6, 33	1.5 [0.4–4.7]	
*TGFB*	-509C > T	C/C (81)	18, 22	Ref	0.7	C/C (47)	14, 30	Ref	0.5
		C/T (60)	13, 22	1.0 [0.4–2.3]		C/T (29)	6, 21	0.5 [0.2–1.7]	
		T/T (13)	4, 31	1.7 [0.4–6.1]		T/T (8)	3, 38	1.3 [0.2–6.2]	
*TLR2*	1350T > C	T/T (133)	33, 25	Ref	0.07	T/T (77)	22, 29	Ref	0.5
		T/C + C/C (21)	2, 10	0.3 [0.04–1.1]		T/C + C/C (7)	1, 14	0.5 [0.02–3.4]	
*TLR4*	896A > G	A/A (137)	31, 23	Ref	0.7	A/A (74)	20, 27	Ref	0.9
		A/G + G/G (17)	4, 24	1.3 [0.3–4.3]		A/G (10)	3, 30	0.9 [0.2–3.8]	
*TNF*	-308G > A	G/G (113)	21, 19	Ref	0.04	G/G (53)	13, 25	Ref	0.5
		G/A + A/A (41)	14, 34	2.4 [1.0–5.7]		G/A + A/A (31)	10, 32	1.4 [0.5–3.8]	

BPAR, biopsy-proven acute rejection; HLA, human leukocyte antigens (HLA-A, -B, and -DR) mismatches; n, number; NA, not available; OR, odds ratio; P, likelihood-ratio P-value; peak PRAs, peak panel-reactive antibodies scores assessed by serum lymphocytotoxicity assay; Ref, reference group; SNP, single nucleotide polymorphism; 95% CI, 95% confidence interval.

Donors*, donor numbers may differ from those in **Table 1**, as each of the 3 deceased donors provided kidneys for 6 different recipients, these 3 donors were counted only once for HWE tests but they were treated independently when associated with BPAR for the individual recipients. In addition, donor numbers may differ within **Table 2** due to genotyping failure.

Recipients^#^, recipient numbers may differ within **Table 1** due to genotyping failure.

In univariate analysis, there was a trend of increasing BPAR incidence for recipient *IL6* -6331T > C (18% in T/T, 25% in T/C, and 46% in C/C; Cochran-Armitage P = 0.031), although it was non-statistically significant after correcting for multiple comparisons (P-value threshold = 0.0036). Similar trends of increasing BPAR incidence were observed in recipient *CRP* -717T > C (16% in T/T, 30% in T/C, and 31% in C/C; Cochran-Armitage P = 0.048), recipient *CASP1* 5352G > A (18% in G/G, 34% in G/A, and 33% in A/A; Cochran-Armitage P = 0.033) and donor *IL6R* -48892A > C (15% in A/A, 28% in A/C, and 47% in C/C; Cochran-Armitage P = 0.019). Point-wise Cochran-Armitage and Fisher’s exact test P-values were > 0.05 for all other recipient and donor SNPs.

**Supplementary Table 2** summarizes recipient and donor genotype differences in BPAR incidence in the first 2 weeks post-transplantation for all 21 SNPs included in the genotyping panel.

## Discussion

To our knowledge, this is the first innate immunogenetic study retrospectively investigating both recipient and donor genetics of pro- and anti-inflammatory mediators for their impact on BPAR incidence in kidney transplant recipients receiving only TAC as the CNI.

The *IL6* -6331 T/T genotype was associated with up to 6-fold higher plasma IL-6 concentrations than C allele carriers in acute inflammatory-status patients post-coronary artery bypass grafting surgery and in patients requiring intensive periodontal therapy, whereas no significant impact was found in healthy volunteers (Smith et al., 2008). However, the relationship between -6331T > C genotypes and plasma IL-6 concentration has not previously been examined post-kidney transplantation, nor the impact of these genotypes on BPAR incidence in kidney transplant recipients. Our results indicate that recipient C/C genotype is associated with 6.6-fold higher odds of BPAR, and with a genotype trend of increasing BPAR incidence from T/T (18%) to T/C (25%) to C/C (46%). However, probably due to a limited sample size (see **Table 2**), the impact of -6331T > C on BPAR incidence was not statistically significant after adjusting for multiple comparisons. Although a recent liver transplant study also failed to show a significant relationship between -6331T > C and BPAR incidence, its sample size was even smaller (liver transplant recipient and donor n = 29; BPAR n = 8), and there were no recipients with the -6331 C/C genotype (Coller et al., 2019). Therefore, the impact of the *IL6* -6331T > C on inflammation and BPAR incidence is still uncertain, and more studies with larger sample sizes are needed to elucidate if this SNP affects BPAR incidence in kidney transplant recipients.

In terms of the impact of *IL2* -330T > G, *IL10* -1082G > A, and *TNF* -308G > A on BPAR incidence, our results are in accordance with previous meta-analyses (Hu et al., 2011; Hu et al., 2015; Xiong et al., 2015; Hu et al., 2016) indicating these SNPs are not significant determinants of BPAR incidence in Caucasian kidney transplant recipients receiving TAC or ciclosporin. Our study also supports cross-sectional studies in which *IL1B* -511C > T did not affect BPAR incidence in kidney transplant recipients receiving TAC or ciclosporin (Marshall et al., 2000; Marshall et al., 2001; Manchanda and Mittal, 2008; Seyhun et al., 2012; Ding et al., 2016). Some studies reported that *IL1B* 3954C > T and *TLR4* 896A > G and 1196C > T affected BPAR incidence but without multiple comparison adjustment (Ducloux et al., 2005; Palmer et al., 2006; Manchanda and Mittal, 2008). These findings were not reproduced in our cohort and in another kidney transplant study exploring the relationship between *TLR4* genetics and BPAR incidence (Nogueira et al., 2007). We are not aware of any other kidney transplant studies investigating the impact of these three SNPs on BPAR incidence in kidney transplant recipients. Recipient and donor *CASP1*, *CRP*, *IL6R*, *MYD88*, and *TLR2* genetics were expected to be important for any innate immune contribution to BPAR incidence in kidney transplant patients, however, common variants in these genes had no significant impact on BPAR incidence in our study. Overall, these results suggest that the innate immunogenetic SNPs investigated (except for *IL6*-6331T > C) are not likely to contribute greatly to BPAR incidence in the first 2 weeks following transplantation in Caucasian kidney transplant recipients receiving immunosuppressive therapy.

Our study has several limitations to consider when interpreting the results. Firstly, as a retrospective study, the limited sample size (recipient and donor n = 151 and 81, respectively) may have been insufficient to support the findings of no major innate immunogenetic impact on BPAR incidence. However, the data presented in this study, along with other innate immunogenetic studies may together provide valuable information for future meta-analyses investigating the impact of innate immunogenetics on BPAR incidence. Secondly, it was necessary to combine some rare homozygous genotypes for statistical purposes; thus the effect of certain rare homozygous genotypes is unknown. Thirdly, some additional SNPs, e.g. *IL6* -174G > C (rs1800795) and *IL10* -592C > A (Lv et al.,2012; Xiong et al.,2015) were not included in this study because of incompatibility with the genotyping array, and insufficient DNA was available to carry out separate genotyping of these SNPs. In addition, other important innate immune genes, e.g. *NFKB1* (encoding for the NF-κB1 subunit) (Misra et al., 2016), were not included in the gene panel design and are worthwhile exploring in the future for their impact on BPAR incidence.

In conclusion, this study did not detect any statistically significant impact of recipient and donor innate immune genetics on BPAR incidence in the first 2 weeks post-kidney transplantation. However, due to the limited sample size, future immunogenetic studies and/or meta-analyses are still required to demonstrate conclusively if innate immune genetics such as *IL6* -6331T > C influence the risk of BPAR incidence post-kidney transplantation.

## Data Availability Statement

The raw data supporting the conclusions of this article will be made available by the authors, without undue reservations, to any qualified researcher.

## Ethics Statement

This study was approved by the Central Adelaide Local Health Network Human Research Ethics Committee (Protocol number 2008178). The patients provided their written informed consent to participate in this study.

## Author Contributions

AS, BS, and JC contributed to the conception and design of the study. JC performed the DNA extraction and collation of genotyping results for the panel. RH and BS collected the clinical dataset. RH and DB performed the statistical analyses. RH wrote the first draft of the manuscript. All authors contributed to manuscript revision, read and approved the submitted version.

## Funding

This study was supported by a project grant (#565038) from the National Health and Medical Research Council, Australia.

## Conflict of Interest

The authors declare that the research was conducted in the absence of any commercial or financial relationships that could be construed as a potential conflict of interest.

## References

[B1] Anders H.-J. Schaefer L. (2014). Beyond tissue injury-damage-associated molecular patterns, toll-like receptors, and inflammasomes also drive regeneration and fibrosis. J. Am. Soc. Nephrol. 25 (7), 1387–1400. 10.1681/ASN.2014010117 24762401 PMC4073442

[B2] ANZDATA Registry (2010). 33rd Report, Chapter 8: Transplantation (Adelaide, Australia: Australia and New Zealand Dialysis and Transplant Registry). https://www.anzdata.org.au/wp-content/uploads/2016/12/Ch08-1.pdf (last accessed on 4th August 2019).

[B3] Awad M. R. El-Gamel A. Hasleton P. Turner D. M. Sinnott P. J. Hutchinson I. V. (1998). Genotypic variation in the transforming growth factor-beta1 gene: association with transforming growth factor-beta1 production, fibrotic lung disease, and graft fibrosis after lung transplantation. Transplantation 66 (8), 1014–1020. 10.1097/00007890-199810270-00009 9808485

[B4] Barratt D. T. Klepstad P. Dale O. Kaasa S. Somogyi A. A. (2015). Innate immune signalling genetics of pain, cognitive dysfunction and sickness symptoms in cancer pain patients treated with transdermal fentanyl. PloS One 10 (9), e0137179. 10.1371/journal.pone.0137179 26332828 PMC4557995

[B5] Coller J. K. Ramachandran J. John L. Tuke J. Wigg A. Doogue M. (2019). The impact of liver transplant recipient and donor genetic variability on tacrolimus exposure and transplant outcome. Br. J. Clin. Pharmacol. 85 (9), 2170–2175. 10.1111/bcp.14034 31219197 PMC6710513

[B6] Coller J. K. White I. A. Logan R. M. Tuke J. Richards A. M. Mead K. R. (2015). Predictive model for risk of severe gastrointestinal toxicity following chemotherapy using patient immune genetics and type of cancer: a pilot study. Support Care Cancer 23 (5), 1233–1236. 10.1007/s00520-014-2481-z 25318697

[B7] Ding S. Xie J. Wan Q. (2016). Association between cytokines and their receptor antagonist gene polymorphisms and clinical risk factors and acute rejection following renal transplantation. Med. Sci. Monit. 22, 4736–4741. 10.12659/msm.898193 27913812 PMC5142584

[B8] Ducloux D. Deschamps M. Yannaraki M. Ferrand C. Bamoulid J. Saas P. (2005). Relevance of Toll-like receptor-4 polymorphisms in renal transplantation. Kidney Int. 67 (6), 2454–2461. 10.1111/j.1523-1755.2005.00354.x 15882292

[B9] Dunning A. M. Ellis P. D. McBride S. Kirschenlohr H. L. Healey C. S. Kemp P. R. (2003). A transforming growth factorbeta1 signal peptide variant increases secretion *in vitro* and is associated with increased incidence of invasive breast cancer. Cancer Res. 63 (10), 2610–2615. 12750287

[B10] Fishman D. Faulds G. Jeffery R. Mohamed-Ali V. Yudkin J. S. Humphries S. (1998). The effect of novel polymorphisms in the interleukin-6 (IL-6) gene on IL-6 transcription and plasma IL-6 levels, and an association with systemic-onset juvenile chronic arthritis. J. Clin. Invest. 102 (7), 1369–1376. 10.1172/JCI2629 9769329 PMC508984

[B11] Galicia J. C. Tai H. Komatsu Y. Shimada Y. Akazawa K. Yoshie H. (2004). Polymorphisms in the IL-6 receptor (IL-6R) gene: strong evidence that serum levels of soluble IL-6R are genetically influenced. Genes Immun. 5 (6), 513–516. 10.1038/sj.gene.6364120 15306846

[B12] Grainger D. J. Heathcote K. Chiano M. Snieder H. Kemp P. R. Metcalfe J. C. (1999). Genetic control of the circulating concentration of transforming growth factor type beta1. Hum. Mol. Genet. 8 (1), 93–97. 10.1093/hmg/8.1.93 9887336

[B13] Hall S. K. Perregaux D. G. Gabel C. A. Woodworth T. Durham L. K. Huizinga T. W. (2004). Correlation of polymorphic variation in the promoter region of the interleukin-1 beta gene with secretion of interleukin-1 beta protein. Arthritis Rheum 50 (6), 1976–1983. 10.1002/art.20310 15188375

[B14] Hammond E. B. Taber D. J. Weimert N. A. Egidi M. F. Bratton C. F. Lin A. (2010). Efficacy of induction therapy on acute rejection and graft outcomes in African American kidney transplantation. Clin. Transplant. 24 (1), 40–47. 10.1111/j.1399-0012.2009.00974.x 19236436

[B15] Hoffmann S. C. Stanley E. M. Darrin Cox E. Craighead N. DiMercurio B. S. Koziol D. E. (2001). Association of cytokine polymorphic inheritance and *in vitro* cytokine production in anti-CD3/CD28-stimulated peripheral blood lymphocytes. Transplantation 72 (8), 1444–1450. 10.1097/00007890-200110270-00019 11685118

[B16] Hu Q. Tian H. Wu Q. Li J. Cheng X. Liao P. (2015). Association between interleukin-2 -330 T/G polymorphism and acute renal graft rejection: a meta-analysis. Transplant. Proc. 47 (6), 1746–1753. 10.1016/j.transproceed.2015.04.090 26293045

[B17] Hu Q. Tian H. Wu Q. Li J. Cheng X. Liao P. (2016). Interleukin-10-1082 G/a polymorphism and acute renal graft rejection: a meta-analysis. Ren Fail 38 (1), 57–64. 10.3109/0886022X.2015.1106770 26524497

[B18] Hu R. Barratt D. T. Coller J. K. Sallustio B. C. Somogyi A. A. (2018). CYP3A5*3 and ABCB1 61A > G significantly influence dose-adjusted trough blood tacrolimus concentrations in the first three months post-kidney transplantation. Basic Clin. Pharmacol. Toxicol. 123 (3), 320–326. 10.1111/bcpt.13016 29603629

[B19] Hu R. Barratt D. T. Coller J. K. Sallustio B. C. Somogyi A. A. (2019a). Effect of tacrolimus dispositional genetics on acute rejection in the first 2 weeks and estimated glomerular filtration rate in the first 3 months following kidney transplantation. Pharmacogenet. Genomics 29 (1), 9–17. 10.1097/FPC.0000000000000360 30489455

[B20] Hu R. Barratt D. T. Coller J. K. Sallustio B. C. Somogyi A. A. (2019b). Is there a temporal relationship between trough whole blood tacrolimus concentration and acute rejection in the first 14 days after kidney transplantation? Ther. Drug Monit 41 (4), 528–532. 10.1097/FTD.0000000000000656 31259882

[B21] Hu X. Bai Y. Li S. Zeng K. Xu L. Liu Z. (2011). Donor or recipient TNF-A -308G/A polymorphism and acute rejection of renal allograft: A meta-analysis. Transpl Immunol. 25 (1), 61–71. 10.1016/j.trim.2011.04.004 21586325

[B22] Kino T. Hatanaka H. Hashimoto M. Nishiyama M. Goto T. Okuhara M. (1987). FK-506, a novel immunosuppressant isolated from a Streptomyces. I. Fermentation, isolation, and physico-chemical and biological characteristics. J. Antibiot (Tokyo) 40 (9), 1249–1255. 10.7164/antibiotics.40.1249 2445721

[B23] Kostura M. J. Tocci M. J. Limjuco G. Chin J. Cameron P. Hillman A. G. (1989). Identification of a monocyte specific pre-interleukin 1 beta convertase activity. Proc. Natl. Acad. Sci. 86 (14), 5227–5231. 10.1073/pnas.86.14.5227 2787508 PMC297594

[B24] Kroeger K. M. Carville K. S. Abraham L. J. (1997). The -308 tumor necrosis factor-alpha promoter polymorphism effects transcription. Mol. Immunol. 34 (5), 391–399. 10.1016/s0161-5890(97)00052-7 9293772

[B25] Lacruz-Guzmán D. Torres-Moreno D. Pedrero F. Romero-Cara P. García-Tercero I. Trujillo-Santos J. (2013). Influence of polymorphisms and TNF and IL1β serum concentration on the infliximab response in Crohn’s disease and ulcerative colitis. Eur. J. Clin. Pharmacol. 69 (3), 431–438. 10.1007/s00228-012-1389-0 22960943

[B26] Li Q. Verma I. M. (2002). NF-kappaB regulation in the immune system. Nat. Rev. Immunol. 2 (10), 725–734. 10.1038/nri910 12360211

[B27] Liew F. Y. Xu D. Brint E. K. O’Neill L. A. (2005). Negative regulation of toll-like receptor-mediated immune responses. Nat. Rev. Immunol. 5 (6), 446–458. 10.1038/nri1630 15928677

[B28] Lim W. H. Chadban S. J. Clayton P. Budgeon C. A. Murray K. Campbell S. B. (2012). Human leukocyte antigen mismatches associated with increased risk of rejection, graft failure, and death independent of initial immunosuppression in renal transplant recipients. Clin. Transplant. 26 (4), E428–E437. 10.1111/j.1399-0012.2012.01654.x 22672477

[B29] Lim W. H. Chapman J. R. Wong G. (2015). Peak panel reactive antibody, cancer, graft, and patient outcomes in kidney transplant recipients. Transplantation 99 (5), 1043–1050. 10.1097/TP.0000000000000469 25539466

[B30] Lv R. Hu X. Bai Y. Long H. Xu L. Liu Z. (2012). Association between IL-6 -174G/C polymorphism and acute rejection of renal allograft: evidence from a meta-analysis. Transpl. Immunol. 26 (1), 11–18. 10.1016/j.trim.2011.10.003 22024650

[B31] Manchanda P. K. Mittal R. D. (2008). Analysis of cytokine gene polymorphisms in recipient’s matched with living donors on acute rejection after renal transplantation. Mol. Cell Biochem. 311 (1–2), 57–65. 10.1007/s11010-007-9694-0 18165865

[B32] Marshall S. E. McLaren A. J. Haldar N. A. Bunce M. Morris P. J. Welsh K. I. (2000). The impact of recipient cytokine genotype on acute rejection after renal transplantation. Transplantation 70 (10), 1485–1491. 10.1097/00007890-200011270-00016 11118095

[B33] Marshall S. E. McLaren A. J. McKinney E. F. Bird T. G. Haldar N. A. Bunce M. (2001). Donor cytokine genotype influences the development of acute rejection after renal transplantation. Transplantation 71 (3), 469–476. 10.1097/00007890-200102150-00022 11233912

[B34] McDonald S. Russ G. Campbell S. Chadban S. (2007). Kidney transplant rejection in Australia and New Zealand: relationships between rejection and graft outcome. Am. J. Transplant. 7 (5), 1201–1208. 10.1111/j.1600-6143.2007.01759 17359502

[B35] Misra M. K. Mishra A. Pandey S. K. Kapoor R. Sharma R. K. Agrawal S. (2016). Association of functional genetic variants of transcription factor Forkhead Box P3 and Nuclear Factor-kappaB with end-stage renal disease and renal allograft outcome. Gene 581 (1), 57–65. 10.1016/j.gene.2016.01.028 26794449

[B36] Mulholland C. V. Somogyi A. A. Barratt D. T. Coller J. K. Hutchinson M. R. Jacobson G. M. (2014). Association of innate immune single-nucleotide polymorphisms with the electroencephalogram during desflurane general anaesthesia. J. Mol. Neurosci. 52 (4), 497–506. 10.1007/s12031-013-0201-7 24352713

[B37] Nankivell B. J. Alexander S. I. (2010). Rejection of the kidney allograft. N Engl. J. Med. 363 (15), 1451–1462. 10.1056/NEJMra0902927 20925547

[B38] Nogueira E. Ozaki K. S. Macusso G. D. Quarim R. F. Camara N. O. Pacheco-Silva A. (2007). Incidence of donor and recipient toll-like receptor-4 polymorphisms in kidney transplantation. Transplant. Proc. 39 (2), 412–414. 10.1016/j.transproceed.2007.01.026 17362744

[B39] Ovsyannikova I. G. Haralambieva I. H. Vierkant R. A. Pankratz V. S. Jacobson R. M. Poland G. A. (2011). The role of polymorphisms in Toll-like receptors and their associated intracellular signaling genes in measles vaccine immunity. Hum. Genet. 130 (4), 547–561. 10.1007/s00439-011-0977-x 21424379 PMC3924423

[B40] Palmer S. M. Burch L. H. Mir S. Smith S. R. Kuo P. C. Herczyk W. F. (2006). Donor polymorphisms in Toll-like receptor-4 influence the development of rejection after renal transplantation. Clin. Transplant. 20 (1), 30–36. 10.1111/j.1399-0012.2005.00436.x 16556150

[B41] Santos-Martins M. Sameiro-Faria M. Ribeiro S. Rocha-Pereira P. Nascimento H. Reis F. (2014). TLR4 and TLR9 polymorphisms effect on inflammatory response in end-stage renal disease patients. Eur. J. Inflammation 12 (3), 521–529. 10.1177/1721727X1401200314

[B42] Saxena V. Lienesch D. W. Zhou M. Bommireddy R. Azhar M. Doetschman T. (2008). Dual roles of immunoregulatory cytokine TGF-β in the pathogenesis of autoimmunity-mediated organ damage. J. Immunol. 180 (3), 1903–1912. 10.4049/jimmunol.180.3.1903 18209088 PMC2291535

[B43] Scheller J. Chalaris A. Schmidt-Arras D. Rose-John S. (2011). The pro- and anti-inflammatory properties of the cytokine interleukin-6. Biochim. Biophys. Acta 1813 (5), 878–888. 10.1016/j.bbamcr.2011.01.034 21296109

[B44] Seyhun Y. Mytilineos J. Turkmen A. Oguz F. Kekik C. Ozdilli K. (2012). Influence of cytokine gene polymorphisms on graft rejection in Turkish patients with renal transplants from living related donors. Transplant. Proc. 44 (5), 1241–1249. 10.1016/j.transproceed.2012.01.125 22663993

[B45] Smith A. J. D’Aiuto F. Palmen J. Cooper J. A. Samuel J. Thompson S. (2008). Association of serum interleukin-6 concentration with a functional IL6 -6331T > C polymorphism. Clin. Chem. 54 (5), 841–850. 10.1373/clinchem.2007.098608 18356242

[B46] Solez K. Colvin R. B. Racusen L. C. Haas M. Sis B. Mengel M. (2008). Banff 07 classification of renal allograft pathology: updates and future directions. Am. J. Transplant. 8 (4), 753–760. 10.1111/j.1600-6143.2008.02159.x 18294345

[B47] Somogyi A. A. Sia A. T. Tan E. C. Coller J. K. Hutchinson M. R. Barratt D. T. (2016). Ethnicity-dependent influence of innate immune genetic markers on morphine PCA requirements and adverse effects in postoperative pain. Pain 157 (11), 2458–2466. 10.1097/j.pain.0000000000000661 27649267

[B48] Taniguchi R. Koyano S. Suzutani T. Goishi K. Ito Y. Morioka I. (2013). Polymorphisms in TLR-2 are associated with congenital cytomegalovirus (CMV) infection but not with congenital CMV disease. Int. J. Infect. Dis. 17 (12), e1092–e1097. 10.1016/j.ijid.2013.06.004 23906542

[B49] Thornberry N. A. Bull H. G. Calaycay J. R. Chapman K. T. Howard A. D. Kostura M. J. (1992). A novel heterodimeric cysteine protease is required for interleukin-1 beta processing in monocytes. Nature 356 (6372), 768–774. 10.1038/356768a0 1574116

[B50] Trompet S. de Craen A. J. Slagboom P. Shepherd J. Blauw G. J. Murphy M. B. (2008). Genetic variation in the interleukin-1 beta-converting enzyme associates with cognitive function. The PROSPER study. Brain 131 (Pt 4), 1069–1077. 10.1093/brain/awn023 18304957

[B51] Turner D. M. Williams D. M. Sankaran D. Lazarus M. Sinnott P. J. Hutchinson I. V. (1997). An investigation of polymorphism in the interleukin-10 gene promoter. Eur. J. Immunogenet. 24 (1), 1–8. 10.1111/j.1365-2370.1997.tb00001.x 9043871

[B52] U. S. Multicenter FK506 Liver Study Group (1994). A comparison of tacrolimus (FK 506) and cyclosporine for immunosuppression in liver transplantation. N. Engl. J. Med. 331(17), 1110–1115. 10.1056/NEJM199410273311702 7523946

[B53] Wadström J. Ericzon B. G. Halloran P. F. Bechstein W. O. Opelz G. Seron D. (2017). Advancing transplantation: new questions, new possibilities in kidney and liver transplantation. Transplantation 101 Suppl 2S, S1–S41. 10.1097/TP.0000000000001563 28125449

[B54] Walsh P. T. Strom T. B. Turka L. A. (2004). Routes to transplant tolerance versus rejection: the role of cytokines. Immunity 20 (2), 121–131. 10.1016/s1074-7613(04)00024-x 14975235 PMC3807577

[B55] Wang L. Lu X. Li Y. Li H. Chen S. Gu D. (2009). Functional analysis of the C-reactive protein (CRP) gene -717A > G polymorphism associated with coronary heart disease. BMC Med. Genet. 10 (1), 73. 10.1186/1471-2350-10-73 19624831 PMC2723087

[B56] Watson J. Mochizuki D. Gillis S. (1980). T-cell growth factors: interleukin 2. Immunol. Today 1 (6), 113–117. 10.1016/0167-5699(80)90047-X 25290353

[B57] Wolf J. Rose-John S. Garbers C. (2014). Interleukin-6 and its receptors: a highly regulated and dynamic system. Cytokine 70 (1), 11–20. 10.1016/j.cyto.2014.05.024 24986424

[B58] Xiong J. Wang Y. Zhang Y. Nie L. Wang D. Huang Y. (2015). Lack of association between interleukin-10 gene polymorphisms and graft rejection risk in kidney transplantation recipients: a meta-analysis. PloS One 10 (6), e0127540. 10.1371/journal.pone.0127540 26035439 PMC4452718

[B59] Zhu L. Fu C. Lin K. Wang Z. Guo H. Chen S. (2016). Patterns of early rejection in renal retransplantation: a single-center experience. J. Immunol. Res. 2016, 2697860. 10.1155/2016/2697860 28058265 PMC5183764

