# Supplementary material for: No Major Effect of Innate Immune Genetics on Acute Kidney Rejection in the First 2 Weeks Post-Transplantation
Source: Front Pharmacol. 2020 Feb 20;10:1686. doi: 10.3389/fphar.2019.01686 (PMC7045476; doi:10.3389/fphar.2019.01686)
Supplement: Supplementary file 1 [file DataSheet_1.docx]

Supplementary Table 1. Recipient and donor *CASP1*, *IL1B*, *IL10* and *TLR4* haplotype and diplotype frequencies

| Genes & SNPs | Recipients^#^ (n = 153 - 154) | | Donors (n = 81) | | | | |
| --- | --- | --- | --- | --- | --- | --- | --- |
|  | Diplotypes (n, %) | Haplotypes (n, %) | Diplotypes (n, %) | | Haplotypes (n, %) | | |
| *CASP1* | G-G/G-G (107, 69)  G-G/A-C (44, 29)  A-C/A-C (3, 2) | G-G (258, 84) | G-G/G-G (58, 72)  G-G/A-C (21, 26)  A-C/A-C (2, 2) | | G-G (137, 85) | | |
| 5352G>A & 10643G>C |  | A-C (50, 16) |  |  | A-C (25, 15) | | |
|  |  |  |  |  |  | | |
| *IL1B* | C-T/C-T (75, 49) | C-T (213, 69) | C-T/C-T (41, 51) | | C-T (114, 70) | | |
| -511C>T & -31T>C | C-T/T-C (63, 41) | T-C (93, 30) | C-T/T-C (32, 40) | | T-C (48, 30) | | |
|  | T-C/T-C (15, 10) | C-C (2, 1) | T-C/T-C (8, 10) | |  | | |
|  | C-C/C-C (1, 1) |  |  | |  | | |
| *IL10* | G-C/G-C (31, 20) | G-C (141, 46) | G-C/G-C (16, 20) | | G-C (68, 42) | | |
|  | G-C/G-T (43, 28) | G-T (89, 29) | G-C/G-T (14, 17) | | G-T (51, 31) | | |
| -1082G>A & -819C>T | G-C/A-T (36, 24) | A-T (76, 25) | G-C/A-T (22, 27) | | A-T (43, 27) | | |
|  | G-T/G-T (14, 9) |  | G-T/G-T (12, 15) | |  | | |
|  | G-T/A-T (18, 12) |  | G-T/-A-T (13, 16) | |  | | |
|  | A-T/A-T (11, 7) |  | A-T/A-T (4, 5) | |  | | |
| *TLR4* | A-C/A-C (136, 88) | A-C (289, 94) | A-C/A-C (71, 88) | | A-C (152, 94) | | |
| 896A>G & 1196C>T | A-C/G-T (16, 10) | G-T (18, 6) | A-C/G-T (10, 12) | | G-T (10, 6) | | |
|  | A-C/A-T (1, 1) | A-T (1, 1) |  | |  | | |
|  | G-T/G-T (1, 1) |  | |  | |  |  |

Percentages may not sum to 100% because of rounding.

n: number; SNP: single nucleotide polymorphism.

Recipients^#^: recipient numbers may differ within this Supplementary Table 1 as one recipient with predicted haplotype probability < 0.8 was not counted.

Supplementary Table 2. Recipient and donor innate immune SNPs and their genotype differences in BPAR incidence in the first 2 weeks post-transplantation

| Genes & SNPs | | Recipients^#^ (n = 153 – 154) | | Donors* (n = 80 – 84) | | |
| --- | --- | --- | --- | --- | --- | --- |
|  |  | Genotypes (n) | BPAR (n, %) | | Genotypes (n) | BPAR (n, %) |
| *BDNF* 196G>A | | G/G (106) | 25, 24 | | G/G (55) | 16, 29 |
|  | | G/A (43) | 9, 21 | | G/A (29) | 7, 24 |
|  | | A/A (5) | 1, 20 | | A/A (0) |  |
| *CASP1* |  | G/G (107) | 19, 18 | | G/G (60) | 16, 27 |
|  | 5352G>A | G/A (44) | 15, 34 | | G/A (22) | 6, 27 |
|  |  | A/A (3) | 1, 33 | | A/A (2) | 1, 50 |
|  |  | G/G (107) | 19, 18 | | G/G (60) | 16, 27 |
|  | 10643G>C | G/C (44) | 15, 34 | | G/C (22) | 6, 27 |
|  | | C/C (3) | 1, 33 | | C/C (2) | 1, 50 |
| *CRP* |  | T/T (77)  T/C (61)  C/C (16) | 12, 16 | | T/T (34)  T/C (39)  C/C (10) | 6, 18 |
| -717T>C | |  | 18, 30 | |  | 15, 38 |
|  | |  | 5, 31 | |  | 2, 20 |
| *IL1B*  -511C>T  -31T>C  3954C>T | | C/C (76)  C/T (63) | 18, 24 | | C/C (41)  C/T (34) | 13, 32 |
|  |  |  | 13, 21 | |  | 9, 26 |
|  |  | T/T (15) | 4, 27 | | T/T (9) | 1, 11 |
|  |  | T/T (74) | 18, 24 | | T/T (41) | 13, 32 |
|  |  | T/C (63) | 13, 21 | | T/C (34) | 9, 26 |
|  |  | C/C (16) | 4, 25 | | C/C (9) | 1, 11 |
|  |  | C/C (84)  C/T (61)  T/T (9) | 16, 19  18, 30  1, 11 | | C/C (54)  C/T (25)  T/T (5) | 13, 24  10, 40  0, 0 |
| *IL2*  -330T>G | | T/T (70)  T/G (63)  G/G (21) | 12, 17 | | T/T (41)  T/G (37)  G/G (6) | 10, 24 |
|  |  |  | 16, 25 | |  | 9, 24 |
|  |  |  | 7, 33 | |  | 4, 67 |
| *IL6*  -6331T>C | | T/T (80)  T/C (61)  C/C (13) | 14, 18 | | T/T (52)  T/C (29)  C/C (3) | 11, 21 |
|  |  |  | 15, 25 | |  | 11, 38 |
|  |  |  | 6, 46 | |  | 1, 33 |
| *IL6R*  48892A>C | | A/A (50)  A/C (78)  C/C (25) | 12, 24 | | A/A (29)  A/C (39)  C/C (15) | 4, 14 |
|  |  |  | 16, 21 | |  | 11, 28 |
|  |  |  | 6, 24 | |  | 7, 47 |
| *IL10*  -1082G>A | | G/G (31)  G/A (79) | 8, 26  19, 24 | | G/G (18)  G/A (37) | 3, 17  11, 30 |
|  | | A/A (43) | 8, 19 | | A/A (29) | 9, 31 |
|  | | C/C (88) | 22, 25 | | C/C (44) | 9, 20 |
| -819C>T | | C/T (54) | 10, 19 | | C/T (36) | 14, 39 |
|  | | T/T (11) | 3, 27 | | T/T (4) | 0, 0 |
| *LY96* | | C/C (146) | 31, 21 | | C/C (79) | 22, 28 |
| 379C>T | | C/T (8) | 4, 50 | | C/T (1) | 0, 0 |
|  | | T/T (0) |  | | T/T (0) |  |
| *MYD88* | | A/A (123)  A/G (29) | 28, 23 | | A/A (66)  A/G (18) | 17, 26 |
| 1593A>G | |  | 7, 24 | |  | 6, 33 |
|  | | G/G (2) | 0, 0 | | G/G (0) |  |
| *OPRM1* | | A/A (115) | 28, 24 | | A/A (55) | 16, 29 |
| 118A>G | | A/G (36) | 7, 19 | | A/G (24) | 6, 25 |
|  | | G/G (3) | 0, 0 | | G/G (1) | 0, 0 |
| *TGFB* | | C/C (81)  C/T (60)  T/T (13) | 18, 22 | | C/C (47)  C/T (29)  T/T (8) | 14, 30 |
| -509C>T | |  | 13, 22 | |  | 6, 21 |
|  |  |  | 4, 31 | |  | 3, 38 |
|  | -1287G>A | G/G (154) | 0, 0 | | G/G (84) | 0, 0 |
| *TLR2* | | T/T (133)  T/C (19) | 33, 25 | | T/T (77)  T/C (6) | 22, 29 |
| 1350T>C | |  | 2, 11 | |  | 1, 17 |
|  |  | C/C (2) | 0, 0 | | C/C (1) | 0, 0 |
| *TLR4* |  | A/A (137)  A/G (16) | 31, 23 | | A/A (74)  A/G (10) | 20, 27 |
|  | 896A>G |  | 4, 25 | |  | 3, 30 |
|  |  | G/G (1) | 0, 0 | | G/G (0) |  |
|  |  | C/C (136) | 30, 22 | | C/C (74) | 19, 26 |
| 1196C>T | | C/T (17) | 5, 29 | | C/T (10) | 3, 30 |
|  | | T/T (1) | 0, 0 | | T/T (0) |  |
| *TNF* -308G>A | | G/G (113)  G/A (35)  A/A (6) | 21, 19  13, 37  1, 17 | | G/G (53)  G/A (30)  A/A (1) | 13, 25  10, 33  0, 0 |

BPAR: biopsy-proven acute rejection; SNP: single nucleotide polymorphism.

Donors*: donor numbers may differ from those in Supplementary Table 1, as each of the 3 donors provided kidneys for 2 different recipients, these 3 donors were counted only once for HWE tests but were treated independently for when associated with BPAR of the individual recipients. In addition, donor numbers may differ within Supplementary Table 2 due to genotyping failure.

Recipients^#^: recipient numbers may differ within Supplementary Table 2 due to genotyping failure.
